# Many pediatric patients with gastroparesis do not receive dietary education

**DOI:** 10.1186/s12876-023-02865-6

**Published:** 2023-07-17

**Authors:** Debra Eseonu, Deepa Dongarwar, Hamisu Salihu, Bruno P. Chumpitazi, Robert J. Shulman

**Affiliations:** 1 Baylor Scott and White Medical Center, 1901 SW H K Dodgen Loop, Temple, TX 76502 USA; 2 University of Texas Medical School at Houston, 6431 Fannin St, Houston, TX 77030 USA; 3 Baylor College of Medicine, 1 Baylor Plaza, Houston, TX 77030 USA; 4 USDA/ARS Children’s Nutrition Research Center, 1100 Bates Ave, Houston, TX 77030 USA

**Keywords:** Child, Diet, Gastroparesis, Education, Nutritionists

## Abstract

**Background:**

Gastroparesis is delayed gastric emptying in the absence of obstruction; dietary modifications are first-line treatment. However, we do not know the factors related to provision of dietary recommendations.

**Methods:**

We sought to determine how often pediatric patients with gastroparesis receive dietary education (from a gastroenterology provider vs dietitian), the recommendations given, and factors related to these outcomes. We performed a retrospective chart review of children 2- to 18-years-old managed by pediatric gastroenterology providers at our institution. Patient demographics and clinical data, dietary advice given (if any), and dietitian consultation (if any), practice location, and prokinetic use were captured. An adjusted binomial regression model identified factors associated with dietary education provision, dietitian consultation, and diet(s) recommended.

**Results:**

Of 161 patients who met criteria, 98 (60.8%) received dietary education and 42 (26.1%) met with a dietitian. The most common recommendation by gastroenterology providers and dietitians was diet composition adjustment (26.5% and 47.6%, respectively). Patients with nausea/vomiting were less likely to receive dietary education or be recommended to adjust diet composition. Patients with weight loss/failure to thrive were more likely to receive dietitian support. Patients seen in the community vs medical center outpatient setting were more likely to be recommended a low-fat diet.

**Conclusions:**

Only a little over half of children with gastroparesis receive dietary education and use of a dietitian’s expertise is much less frequent. Symptoms and clinical setting appear related to what, where, and by whom guidance is provided.

## Introduction

Gastroparesis (Gp) is defined as a delay in the emptying of solids and liquids from the stomach in the absence of a mechanical obstruction [1–3]. Children and adults with Gp commonly report abdominal pain, bloating, early satiety, nausea/vomiting, among other dyspeptic symptoms [1, 2]. Symptom severity can vary; however, symptoms often can be debilitating and lead to significant weight loss and nutritional deficiencies [1, 2]. Symptoms often are associated with food intake [4].

In addition to being a significant burden to patients, Gp also creates a large economic impact on the healthcare system. Between 2004 and 2013, the annual cost of hospitalization for pediatric patients with Gp increased at a rate of $3.4 million/year [5]. Furthermore, the rate of hospitalizations of pediatric patients with Gp in 2013 was 5.2 times greater than in 2004, with patients with Gp having more repeat admissions [5]. Compromised quality of life and the stark increase in healthcare expense emphasize the importance of Gp symptom management by healthcare providers.

The first-line treatment for Gp is dietary management [1–3, 6]. Various diets have been proposed; those frequently recommended in the literature include smaller portion meals, liquid diets, low-fiber diets, and/or low-fat diets [1–3, 6]. For adults there are general guidelines providing guidance on the recommended frequency and general composition of meals; [1–3] but similar published guidelines for pediatric patients are lacking [6]. Importantly, for children with Gp seen in the outpatient setting, it is unknown how often dietary recommendations are given by providers to their patients; which dietary interventions are commonly recommended; if recommendations differ based on patient characteristics or other factors; and if a multidisciplinary approach is used. Our study sought to address these knowledge gaps.

## Methods

A retrospective chart review was conducted to collect pertinent information from visits between pediatric gastroenterology providers (physicians and nurse practitioners) and children with Gp between March 2008 and December 2019. The study was approved by the Baylor College of Medicine Institutional Review Board.

Children with Gp between the ages of 2 and 18 years were included in the study. Patients were classified as having Gp based on gastric scintigraphy results showing greater than 60% gastric retention at the 2-h mark and/or greater than 10% retention at the 4-h mark [1, 2, 7]. Patients were excluded if they had other gastrointestinal comorbidities such as inflammatory bowel disease, celiac disease, eosinophilic esophagitis, peptic ulcer disease, malignancies, or received total parenteral nutrition with no enteral intake. Patients also were excluded if they were diagnosed with Gp but were not seen in an outpatient setting by a pediatric gastroenterology provider.

Patients were identified using The International Classified Diseases, Tenth Revision (ICD-10) codes for the diagnosis of Gp. Additionally, patients were identified by reviewing records of patients undergoing gastric scintigraphy studies where results met the criteria for Gp. Both the ICD-10 codes and scintigraphy logs were collected from an electronic medical records database (EPIC, Verona, WI). Data collected from patient charts included demographics (age, race, ethnicity, gender), and clinical symptom presentation. Failure to thrive and weight loss were based on provider report in the patient chart. The location of the outpatient visit also was noted. The academic setting was defined as outpatient visits conducted at the Texas Children’s Hospital main campus. The community setting was defined as outpatient visits at Texas Children’s Hospital clinics located throughout the greater Houston metropolitan area. Once it was determined that the patient met entry criteria, each outpatient visit was assessed. Dietary data collected included whether dietary recommendations were given; if so, what specific dietary recommendations were provided; and whether a dietitian was consulted. Additionally, for patients who received dietary recommendations, the body mass index (BMI) (kg/m^2^) was determined based on the weight and height of the child measured at the visits. The baseline visit was the visit during which dietary recommendations were provided. The BMI from the last follow-up visit with the gastrointestinal provider was captured to assess change. Prescribed medications, and the timing/sequence of interventions was recorded. All clinic settings had dietitians available onsite for consultation; they regularly worked with pediatric gastroenterology patients and were familiar with dietary recommendations for Gp.

We conducted descriptive statistics to report the demographics and clinical characteristics of the patients. Using Pearson chi-squared test, we conducted bivariate analyses between the patient characteristics and the receipt of dietary recommendations. Next, for the various dietary recommendations provided to the patients, we examined whether the recommendations were given by the pediatric gastroenterology provider and/or the dietitian. Lastly using adjusted logistic regression, we examined the factors associated with each of the following outcomes – dietary education, dietitian consultation, low-fat diet recommendation, small meals recommendation, adjusted meal composition recommendation, recommendation to avoid certain foods, and receipt of a Gp diet handout. The final models for the above associations were chosen based on stepwise function which uses Akaike Information Criterion to select the best model. All statistical analyses were conducted using R version 3∙5∙1 (University of Auckland, Auckland, New Zealand) and R Studio Version 1∙1∙423 (Boston, MA). The type-I error rate was set at 5%.

## Results

A total of 800 patient charts with the diagnostic code of Gp were reviewed. Of these, 161 met inclusion criteria (Table 1). A little more than half the sample were children (versus adolescents). The majority were Non-Hispanic White with approximately 30% of the total being Hispanic. The patient cohort was predominantly female and primarily were seen in the Medical Center outpatient clinic.

**Table 1:** Patient demographics and clinical characteristics (*n* = 161)

	**n (%)**
**Age**	
2–12 years	91 (56.5%)
13- 18 years	70 (43.5%)
**Race/Ethnicity**	
Non-Hispanic White	96 (59.6%)
Non-Hispanic Black	10 (6.2%)
Hispanic	48 (29.8%)
American Indian	1 (0.6%)
Asian	2 (1.2%)
Missing	4 (2.5%)
**Gender**	
Male	57 (35.4%)
Female	104 (64.6%)
**Outpatient Setting**	
Medical Center	140 (87.0%)
Community	21 (13.0%)
**Clinical Presentation**	
Abdominal pain	118 (73.3%)
Nausea and vomiting	97 (60.2%)
Abdominal distension and bloating	9 (5.6%)
Early satiety	10 (6.2%)
Dyspepsia/heartburn and chest pain	9 (5.6%)
Other	95 (59.0%)
2 symptoms	69 (42.8%)
3 symptoms	11 (6.8%)
4 symptoms	1 (0.6%)
**Weight Loss/Failure to Thrive**	
No	105 (65.2%)
Yes	56 (34.8%)
**Route of Nutrition**	
Oral feeds	147 (91.3%)
Oral and enteral tube feeds	11 (6.8%)
All enteral tube feeds	3 (1.9%)
**Prokinetic Medication**	
Total on medication	152 (94.4%)
Macrolide	144 (89.4%)
Metoclopramide	17 (10.6%)
Bethanechol	33 (20.5%)
Other	13 (8.1%)

When assessing clinical presentation based on cardinal Gp symptoms [3, 6, 8], abdominal pain was the most common complaint, followed by nausea and vomiting (Table 1). Most patients did not have weight loss or failure to thrive. The most prominent route of nutrition was oral. A large majority of the patients were prescribed prokinetic medications, with the most common being macrolides. Based on the calculated gastric retention at the 4-h timepoint of the gastric scintigraphy study, of the 161 patients who met inclusion criteria, 5 (3.1%) had less than 10% delay in gastric emptying, 60 (37.2%) had 10–20% delay, 39 (24.2%) had 20–30% delay, and 57 (35.4%) had greater than 30% delay.

Only 98 (60.8%) of the 161 patients in the study received dietary education during an outpatient visit. Of these 28 (28.6% of the total group) received dietary education from both the pediatric gastroenterology provider and dietitian; 56 (57.1% of the total group) received dietary education only from the pediatric gastroenterology provider; and 14 (14.3% of the total group) received dietary education only from the dietitian.

Patient demographics and clinical characteristics were evaluated against the frequency of dietary education (Table 2). However, there were no associations between the provision of dietary education and age, race, ethnicity, gender, outpatient setting, cardinal Gp symptoms, weight loss/failure to thrive, or route of nutrition.

**Table 2:** Patient Characteristics by Whether or not Dietary Education was Given

	**No Dietary Education**	**Dietary Education**	***P*-value**
	***n* = 63**	***n* = 98**	
**Age**			0.65
2–12 years	37 (58.7%)	54 (55.1%)	
13- 18 years	26 (41.3%)	44 (44.9%)	
**Race/Ethnicity**			0.81
Non-Hispanic White	40 (63.5%)	56 (57.1%)	
Non-Hispanic Black	4 (6.3%)	6 (6.1%)	
Hispanic	16 (25.4%)	32 (32.7%)	
Other/Missing^a^	3 (4.8%)	4 (4.1%)	
**Gender**			0.81
Male	23 (36.5%)	34 (34.7%)	
Female	40 (63.5%)	64 (65.3%)	
**Outpatient Setting**			0.92
Medical Center	55 (87.3%)	85 (86.7%)	
Community	5 (7.9%)	13 (13.3%)	
**Two Cardinal Symptoms:**			
Abdominal pain and nausea/vomiting	25 (39.7%)	31 (31.6%)	0.29
Abdominal pain and early satiety	1 (1.6%)	1 (1.0%)	0.75
Nausea/vomiting and early satiety	0 (0.0%)	3 (3.1%)	0.27
Nausea/vomiting and dyspepsia	2 (3.2%)	0 (0.0%)	0.17
Nausea/vomiting and abdominal distension	1 (1.6%)	0 (0.0%)	0.41
Abdominal pain and dyspepsia	0 (0.0%)	2 (2.0%)	0.25
Abdominal pain and distension	0 (0.0%)	2 (2.0%)	0.35
**Weight Loss/Failure to Thrive**			0.16
No	46 (73.0%)	60 (61.2%)	
Yes	17 (27.0%)	38 (38.8%)	
**Route of nutrition**			0.31
Full oral	55 (87.3%)	91 (92.9%)	
Oral and enteral tube feeds	7 (11.1%)	4 (4.1%)	
All enteral tube feeds	1 (1.6%)	2 (2.0%)	

^a^Other/missing includes American Indian, Asian, and those without racial/ethnicity demographics listed. Due to the small number of patients these were analyzed as a group

**Fig. 1:**
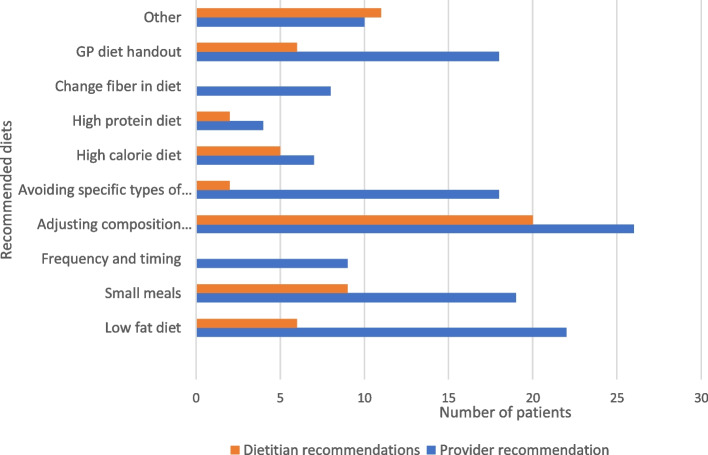
Diet recommendations by physicians and/or dietitian. Orange bars refer to dietitian recommendations, blue bars refer to provider recommendations

When a dietary recommendation was given, the most common was adjusting the diet composition (26.5%) (Fig. 1). This was defined as either switching to a full liquid diet, supplementation with an enteral formula, or use of a pureed diet. Additionally, a low-fat diet (22.4%) and small meals (19.4%) frequently were recommended. When dietitians were consulted, the most frequent recommendation also was adjusting the diet composition (47.6%).

A total of 46 patients (28.6%) were given dietary education prior to starting prokinetic medications. A similar number (43 patients, 26.7%) received dietary education and initiation of prokinetic medication simultaneously. Approximately 19 patients (11.8%) were started on prokinetic medication prior to receiving dietary education.

Odds ratios were used to determine the likelihood of receiving any dietary education, consultation with a dietitian, and a specific dietary adjustment being recommended (Table 3). Weight loss and/or failure to thrive were associated with a greater likelihood of receiving dietary recommendations from a dietitian(3.17 [95% CI: 1.41–7.35]). Small meals were more likely to be recommended in patients with abdominal pain (3.59 [95%CI: 1.12–7.24]). Finally, a low-fat diet was more likely to be recommended in a community clinic (5.26 [95% CI: 1.68–16.28]).

**Table 3:** Factors associated with various outcomes [Odds Ratio (95% CI)]

	**Dietary Education**	**Dietitian Consultation**	**Low Fat Diet Recommendation**	**Small Meals Recommendation**	**Adjusted Composition**
**Age**					
0–12 years	─^b^	─	─	─	─
13-above:	─	─	─	─	─
**Race/ethnicity**					
NH-White	─	ref^a^	─	─	─
NH-Black	─	0.17 (0.01–1.08)	─	─	─
Hispanic	─	0.56 (0.23–1.28)	─	─	─
Others	─	─	─	─	─
**Outpatient Setting**					
Academic	–	ref	ref	ref	–
Community	–	**0.13 (0.02–0.06)**	**5.26 (1.68–16.28)**	2.16 (0.72–10.59)	–
**Cardinal symptoms**					
Abdominal pain	–	–	–	**3.59 (1.12–7.24)**	–
Nausea and vomiting	**0.46 (0.23–0.91)**	–	–	–	**0.42 (0.17–0.97)**
**Weight loss/Failure to thrive**					
No	ref	ref	ref	ref	ref
Yes	1.72 (0.86- 3.51)	**3.17 (1.41–7.35)**	0.38 (0.11–1.09)	0.54 (0.14–1.68)	1.28 (0.49- 3.18)
**Route of nutrition**					
Full oral	ref	–	–	ref	ref
Oral and tube feedings	**0.26 (0.05–0.97)**	–	–	–	1.98 (0.13- 6.63)
All tube feedings	1.31 (0.12- 2.98)	–	–	2.38 (0.17- 25.21)	4.05 (0.17- 6.87)

Text in Bold represent statistically significant findings

*Abbreviations*: *NH-Black* Non- Hispanic Black, *NH-White* Non-Hispanic White

^a^Reference category for association models

^b^The covariates selected were determined using a stepwise function which uses Akaike Information Criterion (AIC) to select the best model

In contrast, nausea and vomiting and the use of oral and tube feedings were associated with less likelihood that dietary education would be provided (0.46 [95% CI: 0.23–0.91]), (0.26 [95%CI: 0.05–0.97], respectively). Similarly, adjustment of meal composition was less likely to be recommended in patients with nausea and vomiting (0.42 [95%CI: 0.17–0.97]). In addition, being seen in a community clinic made it less likely that the patient would see a dietitian (0.13 [95% CI: 0.02–0.06]). Age, race, or ethnicity were not associated with dietary education, consultation with a dietitian, or modification of the diet.

Because the BMI data was not normally distributed, median with minimum and maximum ranges are shown (Table 4). Of the 98 subjects who received dietary education, 91 had follow-up visits and 7 were seen only once in clinic. In the no-dietary education group, 62 out of the 63 subjects had follow-up visits.

**Table 4:** Changes in Body Mass Index (BMI)

Dietary Education	Baseline BMI	Last Clinic Visit BMI	Median Change in BMI	Time Between Visits (months)^b^	Change in BMI per Month	Change in BMI *P* Value	Change in BMI per Month between Groups *P* Value
Yes (*n* = 91)	17.9 (11.8–38.0)^a^	19.7 (11.1–47.3)	1.0 (-5.7–20.0)	26.0 (1.0–109.0)	0.06 (-0.9–0.5)	< 0.001	0.22
No (*n* = 62)	17.1 (13.7–31.5)	19.2 (14.2–37.0)	0.6 (-3.8–7.9)	25.0 (1.0–119.0)	0.03 (-0.9–0.5)	0.001	

^a^Minimum—Maximum

^b^Time between visits (months) refers to time from first visit to the pediatric GI clinic to the last visit recorded for this study

The increase in BMI was significant for both the dietary education group (*P* < 0.001) and the no-dietary education group (*P* = 0.001) taking into account the time between baseline and final clinic visit. Although the increase in BMI was numerically greater for the dietary education group, the difference between groups did not reach significance (*P* = 0.22).

## Discussion

Dietary modification is the first-line treatment for Gp and should be utilized by healthcare providers when working towards improving symptoms [1–3, 6]. However, a substantial minority of patients with Gp at our center (41%) did not receive any dietary education. Only 26% of patients had a consultation with a dietitian. We identified that clinical symptoms (e.g., weight loss, vomiting/nausea) and practice location (medical center vs suburban) were associated with whether dietary education was provided and the type of dietary recommendation given. Our findings suggest there appears to be a large need to increase the amount and type of dietary education provided to children with Gp if our findings reflect what is occurring nationally.

Clinical guidelines for managing Gp, focused on adults, start with dietary recommendations [1–3]. In contrast, a large proportion of the children in our study were prescribed prokinetic medications before dietary education was provided. Use of dietary management has the potential benefit of providing patients and families with more autonomy by allowing them greater choice of food and food types to help address symptoms, while also avoiding potential significant side effects associated with medications. When and why dietary recommendations versus medications should be recommended based on symptoms requires further study.

The dietary recommendations, when they were provided, were in line with Gp treatment guidelines. Specifically, the guidelines recommend that meals be small, and patients eat more frequently (4–5 times a day) to reach their required caloric intake [9]. The rationale behind smaller meals is that the stomach empties at a constant rate, estimated at 1–2 kcal/min, so larger size meals with more caloric content take longer to empty [10]. Symptomatically, large meals also increase intragastric pressure, which can worsen the baseline symptoms patients are experiencing [9].

Foods that are blended, mashed, or ground as well as high calorie liquids are an encouraged alternative to solid meals [9]. As an example, on a diet consisting of small particle-sized solids, reflux symptoms, fullness, nausea, vomiting and bloating (but not abdominal pain) improved in a randomized, controlled study in patients with diabetic Gp [11].

Liquid diets may be used because typically patients diagnosed with delayed gastric emptying of solid foods have preserved liquid emptying [9, 12]. As noted above, dietary modification has the benefit of providing patients and families with the autonomy to choose foods within specific categories, potentially without the need for medications.

A low fat, low-fiber diet also is recommended [9]. Use of low-fat diets is predicated on the ileal brake mechanism; macronutrients, particularly fats, when reaching the ileum generate a hormonal negative feedback loop that delays gastric emptying and decreases duodenal/jejunal motility; this may lead to decreased intake [12, 13]. The recommendation to decrease fiber intake is based on the idea that less fiber makes it easier for the stomach to produce chyme and pass it into the duodenum [9, 12]. Additionally, fiber consumption can lead to phytobezoar production [9].

In contrast with adult guidelines which first recommend adjusting meal size [9], the most common dietary recommendation given by the providers and dietitians was adjusting the composition of the meal. Adjusting meal size was the second most common recommendation by the dietitians and the third most common recommendation by the providers.

A novel finding in our study was that clinical symptoms appeared to be a driving force in whether dietary recommendations were provided, a dietitian consulted, and the dietary changes recommended. Patients with nausea and vomiting were less likely to receive dietary education or be recommended to modify their diet composition. We speculate that nausea and/or vomiting symptoms may have prompted the pediatric gastroenterology providers to prescribe medication rather than pursue diet changes. Indeed, published consensus guidelines for the management of Gp in adults encourage the use of medication for nausea and vomiting [1, 3]. Although anti-nausea medications can be helpful, studies also have shown improvement in nausea and vomiting after dietary modifications [11].

Patients who exhibited weight loss/failure to thrive were more likely to have a consultation with a dietitian. This is not surprising as pediatric providers (not just in gastroenterology) commonly look to dietitians for assistance with these conditions. Pediatric providers in general are keenly attuned to the dangers of weight loss and failure to thrive in terms of impairing normal growth and development.

Patients managed in the community outpatient setting were less likely to consult with a dietitian; reason(s) for which are unclear. This was not due to clinic infrastructure as the community outpatient clinics have same resources (including dietitians) as the medical center outpatient clinic. It is possible that patient factors (e.g., socioeconomic differences) or provider factors (e.g., more knowledge of diets for Gp) played a role. Conversely, patients receiving partial tube feeding were less likely to receive dietary education. It may be an indication that parents who have dealt with tube feeding already are well-versed in this type of nutritional management because of previous input from dietitians. Dietitians are skilled at balancing appropriate intake based on what patients can consume orally and what will be given enterally [14]. Moreover, they commonly teach families enteral formula preparation.

There was a low prevalence of weight loss/failure to thrive in the pediatric patients with Gp assessed in this study, which is a novel finding. These data are consistent with the data in adults with Gp, where only 10% were identified as being underweight [15]. In parallel with adults with Gp, our cohort of children with Gp are generally not malnourished but may have compromises in their nutritional intake that may negatively impact their health. When following the BMI changes in patients from their baseline to their last clinic visit with pediatric gastroenterologists, both those who did and did not receive dietary education had improvements in their BMI that were significant. Though both groups showed improvements in their BMI, there was no statistically significant difference when comparing groups. The lack of significance may be due to variability within the retrospective study. That said, the trend of the data shows the group who received dietary education had a larger improvement in BMI overall and in the change per month. Prospective controlled studies are necessary to address a potential impact of dietary education on change in BMI.

There are some limitations to our study. Being retrospective, data extracted was based on what was documented in the chart. There may have been discussions with the pediatric gastroenterology provider or dietitian that were not recorded. However, it is standard clinical practice to document recommendations in the medical chart, particularly when treatment-related recommendations are made. Due to the retrospective nature of the study, investigating possible relationships between the patients’ primary symptoms and the use of dietary intervention was not possible. Given a lack of documentation and standardization of reporting, assessing pain and nausea/vomiting as well as severity of symptoms was not possible. This limited the ability to investigate possible correlations between dietary interventions and symptoms in this study but would be beneficial to pursue in future prospective studies. Additionally, there is the possibility that patients with Gp may have utilized supportive healthcare providers like dietitians from within the community, rather than from our institution; however, in our experience this is rare. The generalizability of the data may be limited given the inclusion of only one healthcare system. However, the number of pediatric gastroenterology providers in our healthcare system managing patients with Gp is large (> 35) and encompass a broad range of educational backgrounds and clinical experience. Moreover, Houston is one of the most racially and ethnically diverse cities in the US which is likely to contribute to the generalizability of our findings. We hope to emphasize the above critical gaps in the literature for others to develop controlled studies to assess clinical outcomes related to dietary therapy and Gp in pediatric patients.

Strengths of the study include the relatively large sample size and the availability of a dietitian in all clinics. In addition, all included patients underwent the recommended testing to confirm a diagnosis of Gp. Another strength is that our findings were obtained from patients during routine clinical practice in a variety of practice settings, increasing the generalizability of the results.

In summary, our study highlights the need for greater use of dietary education as first-line therapy in children with Gp—congruent with current treatment guidelines for patients with this disorder. Further, greater use of dietitian consultation is likely warranted. Future efforts may include the implementation of strategies to increase awareness of the need for dietary counseling among providers caring for children with Gp, and further investigation of the efficacy of dietary therapies in the setting of varied Gp-associated symptoms through the assessment of clinical outcomes.

## Data Availability

All data generated or analysed during this study are included in this published article.

## Abbreviations

Gp: Gastroparesis
BMI: Body mass index

## References

[1] Lacy BE, Tack J, Gyawali CP (2022). AGA clinical practice update on management of medically refractory gastroparesis: expert review. Clin Gastroenterol Hepatol.

[2] Camilleri M, Kuo B, Nguyen L, Vaughn VM, Petrey J, Greer K (2022). ACG Clinical Guideline: Gastroparesis. Am J Gastroenterol.

[3] Schol J, Wauters L, Dickman R, Drug V, Mulak A, Serra J (2021). United European Gastroenterology (UEG) and European Society for Neurogastroenterology and Motility (ESNM) consensus on gastroparesis. Neurogastroenterol Motil.

[4] Wytiaz V, Homko C, Duffy F, Schey R, Parkman HP (2015). Foods provoking and alleviating symptoms in gastroparesis: patient experiences. Dig Dis Sci.

[5] Lu PL, Moore-Clingenpeel M, Yacob D, Di Lorenzo C, Mousa HM (2016). The rising cost of hospital care for children with gastroparesis: 2004–2013. Neurogastroenterol Motil.

[6] Febo-Rodriguez L, Chumpitazi BP, Shulman RJ (2020). Childhood gastroparesis is a unique entity in need of further investigation. Neurogastroenterol Motil.

[7] Abell TL, Camilleri M, Donohoe K, Hasler WL, Lin HC, Maurer AH (2008). Consensus recommendations for gastric emptying scintigraphy: a joint report of the American Neurogastroenterology and Motility Society and the Society of Nuclear Medicine. Am J Gastroenterol.

[8] Febo-Rodriguez L, Chumpitazi BP, Musaad S, Sher AC, Shulman RJ (2021). Meal-induced symptoms in children with dyspepsia-relationships to sex and the presence of gastroparesis. J Pediatr.

[9] Limketkai BN, LeBrett W, Lin L, Shah ND (2020). Nutritional approaches for gastroparesis. Lancet Gastroenterol Hepatol.

[10] Meyer JH, Ohashi H, Jehn D, Thomson JB (1981). Size of liver particles emptied from the human stomach. Gastroenterology.

[11] Olausson E, Storsrud S, Grundin H, Mag P, Isaksson M, Attvall S (2014). A small particle size diet reduces upper gastrointestinal symptoms in patients with diabetic gastroparesis: a randomized controlled trial. Am J Gastroenterol.

[12] Hellstrom PM, Gryback P, Jacobsson H (2006). The physiology of gastric emptying. Best Pract Res Clin Anaesthesiol.

[13] Shin H, Ingram J, McGill A, Poppitt S (2013). Lipids, CHOs, Proteins: Can All Macronutrients Put a ‘Brake’ on Eating?. Physiol Behav.

[14] Edwards S, Davis A, Bruce A, Mousa H, Lyman B, Cocjin J (2016). Caring for tube-fed children: a review of management, tube weaning, and emotional considerations. J Parenter Enter Nutr.

[15] Parkman HP, Van Natta M, Yamada G, Grover M, McCallum RW, Sarosiek I (2021). Body weight in patients with idiopathic gastroparesis. Neurogastroenterol Motil.

